# Editorial: Research in obesity, type 2 diabetes, and metabolic syndrome: cellular pathways and therapeutic innovations

**DOI:** 10.3389/fcell.2026.1849892

**Published:** 2026-05-01

**Authors:** Mayank Choubey, Rashu Barua, Munichandra Babu Tirumalasetty, Md Wahiduzzaman, Mohammad Sarif Mohiuddin

**Affiliations:** Department of Foundations of Medicine, NYU Grossman Long Island School of Medicine, Mineola, NY, United States

**Keywords:** adiposity, chronic diseases, MASH, MASLD, metabolic syndrome, obesity, type 2 diabetes

Obesity, T2DM, and metabolic syndrome remain major and rapidly growing global public health challenges, with obesity rising worldwide, 589 million adults currently living with diabetes globally, and metabolic syndrome prevalence increasing substantially over time (World Health Organization, 2025; International Diabetes Federation, 2025; Noubiap et al., 2025). These interconnected disorders are associated with substantial morbidity and mortality and contribute to a broad spectrum of complications, including cardiovascular disease, metabolic dysfunction-associated steatotic liver disease (MASLD), reproductive dysfunction, neurobehavioral alterations, and progressive end-organ injury. Although their clinical burden is well established, the underlying biology is highly complex, involving chronic nutrient excess, insulin resistance, adipose tissue dysfunction, inflammation, oxidative stress, altered lipid handling, gut and microbial influences, mitochondrial perturbation, and multi-organ crosstalk. Continued progress in this field depends on studies that not only refine our understanding of pathophysiological mechanisms but also identify clinically useful biomarkers and novel therapeutic opportunities.

1. This Research Topic, Research in Obesity, Type 2 Diabetes, and Metabolic Syndrome: Cellular Pathways and Therapeutic Innovations, was assembled to highlight recent advances across these interconnected areas of metabolic disease. The Research Topic brings together review articles and original investigations addressing MASLD, insulin resistance, biomarker discovery, metabolic heterogeneity, neurobehavioral regulation, metabolic syndrome, and translational risk assessment. Together, these contributions reinforce the concept that obesity-related disease is not a single-organ disorder, but rather a systemic condition driven by interacting molecular, cellular, and physiological networks.
2. A major theme emerging from this Research Topic is the growing centrality of MASLD within the broader landscape of metabolic disease. Liu et al. reviewed how disrupted organelle interaction networks contribute to the pathogenesis and therapy of MASLD, emphasizing impaired communication among mitochondria, endoplasmic reticulum, lipid droplets, and autophagic systems. Their work moves the field beyond traditional descriptions of hepatic lipid accumulation and frames MASLD as a disorder of impaired cellular coordination and reduced metabolic flexibility. Complementing this mechanistic perspective, Mohiuddin et al. summarized the bidirectional links between MASLD and T2DM, highlighting shared drivers such as insulin resistance, lipogenesis, inflammatory signaling, and microbiota-related alterations, while also discussing the clinical implications of this increasingly common coexistence. Together, these articles provide an integrated framework for understanding MASLD as both a manifestation and a driver of systemic metabolic dysfunction.
3. Several original studies in this Research Topic further address the need for improved clinical tools to identify MASLD and its complications in high-risk populations. Wang W. et al.identified the hypersensitive C-reactive protein-atherogenic index as a potential marker of MASLD in T2DM, suggesting improved diagnostic utility compared with several conventional metabolic indices. Guo et al. reported that the serum uric acid to apolipoprotein A1 ratio is independently associated with MASLD in T2DM, further supporting the value of composite circulating biomarkers in metabolic liver disease risk assessment. Al-Ajmi et al. added an anthropometric dimension by showing that wrist circumference is associated with MASLD in the KADEM cohort, suggesting that simple body measurements may aid screening in routine practice. Yuan et al. further demonstrated that the triglycerides/high-density lipoprotein-cholesterol ratio outperforms traditional lipid indicators in predicting MASLD among U.S. adults, supporting the clinical utility of accessible lipid-based markers in disease risk stratification. Extending this focus from steatosis to disease progression, Tang et al. showed that inflammatory markers may predict liver fibrosis in T2DM patients with MASLD, emphasizing the translational value of inflammation-based indices in disease monitoring and stratification. Collectively, these studies highlight the ongoing shift toward practical, scalable, and clinically accessible approaches to metabolic liver disease detection and progression assessment.
4. This Research Topic also captures the diversity of metabolic liver injury and related molecular mechanisms beyond classic non-alcohol-related phenotypes. Song C. et al. provided systems-level metabolic insight into kaempferol-mediated alleviation of alcoholic fatty liver disease, underscoring how pathway-oriented analyses can reveal therapeutic mechanisms relevant to hepatic metabolic injury. Although alcoholic fatty liver disease differs from MASLD in etiology, the study broadens the discussion of hepatic metabolic stress, redox imbalance, and intervention strategies within the wider spectrum of steatotic liver disorders. In addition, Baidya et al. showed that METTL3-mediated activation of Sonic Hedgehog signaling promotes breast cancer progression, extending the Research Topic toward broader cell signaling and epigenetic regulatory mechanisms relevant to disease-associated cellular remodeling.
5. Another important thread across the Research Topic is the investigation of insulin resistance as a central mechanistic hub linking obesity, dyslipidemia, visceral adiposity, and diabetes risk. In a systematic review, Song Y. et al. showed that the rs7799039 variant in the leptin gene promoter is associated with reduced serum leptin levels and higher insulin levels, supporting a genetic contribution to insulin resistance through altered leptin biology. In a large clinical study, Chen et al. examined the relationship between *Helicobacter pylori* infection and visceral fat area in diabetic populations and identified insulin resistance as a mediating factor, thereby linking infection-related influences to metabolic adiposity. From a population-level perspective, Ito et al. used energy landscape analysis to identify distinct pathways to diabetes development in obese and non-obese individuals, showing that progression toward diabetes does not follow a single uniform route. Together, these studies underscore the heterogeneity of metabolic disease while positioning insulin resistance as a shared axis through which diverse genetic, infectious, and physiological factors converge.
6. The Research Topic also broadens the discussion of metabolic disease beyond the liver and conventional glycemic endpoints. Gao et al. investigated appetite-related alterations in overweight and obese patients with T2DM using multimodal magnetic resonance imaging and identified structural and functional changes in brain regions linked to ingestive desire. In an experimental model, Rodrigues et al. examined serotoninergic modulation in the brainstem and hypothalamus of female overnourished rats and demonstrated effects on mitochondrial markers, oxidative stress, and BDNF mRNA levels. Wang B. et al. reported a curvilinear relationship between Life’s Crucial 9 and metabolic syndrome in U.S. adults, suggesting that healthier composite lifestyle and cardiometabolic profiles are associated with lower metabolic syndrome burden. Reproductive consequences of metabolic dysfunction were addressed by Ma and Xi, who investigated the association between metabolic syndrome and sperm DNA fragmentation index in a regional multicentre prospective long-term follow-up study, highlighting the broader systemic effects of metabolic disease in men of reproductive age. Together, these findings reinforce that obesity, T2DM, and metabolic syndrome extend well beyond traditional cardiometabolic endpoints and influence neural, behavioral, and reproductive health.
7. Overall, the articles collected in this Research Topic provide a multidimensional view of obesity, T2DM, and metabolic syndrome, spanning organelle biology, hepatic disease mechanisms, biomarker development, insulin resistance, neurobehavioral regulation, reproductive health, and metabolic systems modeling. A unifying message across these contributions is that metabolic disease is driven by dynamic interactions among cellular stress pathways, inflammatory and endocrine signals, tissue remodeling, behavioral regulation, and organ crosstalk. The work presented here advances both mechanistic understanding and translational insight, particularly in the areas of MASLD pathogenesis, risk stratification, fibrosis prediction, insulin resistance, and metabolic heterogeneity.

As Research Topic Editors, we sincerely thank all authors for their valuable contributions, the reviewers for their rigorous evaluations and constructive feedback, and the editorial staff for their support throughout the development of this Research Topic. We hope this Research Topic will serve as a useful resource for investigators and clinicians seeking to better understand the biological complexity of obesity-related metabolic disorders and to develop more precise and effective strategies for prevention, diagnosis, and therapy. This Research Topic, *Research in Obesity, Type 2 Diabetes, and Metabolic Syndrome: Cellular Pathways and Therapeutic Innovations*, was assembled to highlight recent advances across these interconnected areas of metabolic disease. The Research Topic brings together review articles and original investigations addressing MASLD, insulin resistance, biomarker discovery, metabolic heterogeneity, neurobehavioral regulation, and translational risk assessment. Together, these contributions reinforce the concept that obesity-related disease is not a single-organ disorder, but a systemic condition driven by interacting molecular, cellular, and physiological networks.

## References

[B1] International Diabetes Federation (2025). IDF diabetes atlas. 11th ed. Brussels: International Diabetes Federation.

[B2] Noubiap J. J. Nansseu J. R. Nyaga U. F. Ndoadoumgue A. L. Ngouo A. T. Tounouga D. N. (2025). Worldwide trends in metabolic syndrome from 2000 to 2023: a systematic review and modelling analysis. Nat. Commun. 17, 573. 10.1038/s41467-025-67268-5 41350289 PMC12808104

[B3] World Health Organization (2025). Obesity and overweight. Geneva: World Health Organization.

